# Association between chronic disease multimorbidity and leisure-time physical activity: Evidence from the China Multiethnic Cohort study

**DOI:** 10.3389/fmed.2022.874456

**Published:** 2022-07-27

**Authors:** Yajie Li, Xianzhi Li, Bin Yu, Jingzhong Li, Ruifeng He, Qucuo Nima, Junmin Zhou

**Affiliations:** ^1^Tibet Center for Disease Control and Prevention, Lhasa, China; ^2^Clinical Research Center, Panzhihua Central Hospital, Panzhihua, China; ^3^Meteorological Medical Research Center, Panzhihua Central Hospital, Panzhihua, China; ^4^Institute for Disaster Management and Reconstruction, Sichuan University - Hong Kong Polytechnic University, Chengdu, China; ^5^West China School of Public Health and West China Fourth Hospital, Sichuan University, Chengdu, China

**Keywords:** chronic diseases, multimorbidity, leisure-time physical activity, ethnic differences, system

## Abstract

**Objective:**

To reveal the associations between multimorbidity and leisure-time physical activity (LTPA) by ethnicities in China.

**Materials and methods:**

Self-reported information on a range of occupational, household, transport, and LTPA was collected by interviewer-administered questionnaire. A total of 17 chronic diseases were assessed based on self-reported lifetime diagnoses or medical examinations. Multivariable logistic regression models were used to assess the associations between multimorbidity and the risks of low LTPA.

**Results:**

The mean age of all participants was 51.2 years old. Of all, 61.4% were women and 57.9% were from the Han population. A significantly negative association (OR = 0.92, 95% CI = 0.89–0.95) was found between multimorbidity and low LTPA, with a stronger association among minority populations (OR = 0.86, 95% CI = 0.82–0.91) than among the Han population (OR = 0.96, 95% CI = 0.92–1.01). For both the minority population and the Han population, digestive system multimorbidity and digestive-metabolic system multimorbidity had a significantly negative association with low LTPA. For the Han population, the association of intersystem multimorbidity for the circulatory-respiratory system (OR = 1.17, 95% CI = 1.04–1.31) with low LTPA was stronger than that of intrasystem multimorbidity for the circulatory (OR = 1.12, 95% CI = 1.01–1.25) and respiratory systems (OR = 1.14, 95% CI = 1.04–1.25).

**Conclusion:**

There are significant associations between multimorbidity and low LTPA based on this large multiethnic population. Our findings suggest that LTPA-tailored interventions should be designed for specific ethnic groups according to different types of multimorbidity.

## Introduction

Multimorbidity, a term commonly used to describe the presence of two or more chronic physical conditions (1), has been widely acknowledged as a public health challenge. Despite the inconsistency in measurement, the prevalence of multimorbidity is ranging from 30% to 95% across age groups and countries (2, 3). People with multimorbidity suffer more pain, mobility limitations, and higher mortality (4, 5), and secondary prevention of a wide range of chronic diseases deserves attention in this population. To slow chronic disease progression and reduce mortality, regular physical activity (PA) has been recommended by the WHO in the multifaceted care of individuals with chronic disease or multimorbidity (6, 7). At the macro level, PA helps to reduce the disease burden and loss of economic output associated with the treatment of chronic disease complications, especially in low- and middle-income countries (LMICs) (8).

Evidence exists about the different levels of PA in people living with multimorbidity. For example, a study conducted on 46 LMICs revealed that people with chronic conditions and multimorbidity were significantly less physically active (9). Another study conducted on 96,706 United Kingdom Biobank participants (aged 40 years old or older) suggested that participants with chronic disease undertook 9% or 61 min less moderate activity and 11% or 3 min less vigorous activity per week than individuals without chronic disease (10). However, limited studies clarify whether and to what extent multimorbidity is associated with leisure-time physical activity (LTPA), the most important domain of PA (11). Moreover, multimorbidity of chronic disease may be present in multiple system groups (e.g., cardiorespiratory system (12) and psychiatric system (13)), and therefore usually refers to systematic multimorbidity (i.e., intersystem multimorbidity and intrasystem multimorbidity). Understanding the level of LTPA in people living with intersystem or intrasystem multimorbidity can help clinicians for better guidance on rehabilitation exercises to reduce the risk of complications, disability, and death, but such evidence is currently lacking.

Numerous studies have explored racial and ethnic disparities in PA worldwide (14, 15) and found racial/ethnic minorities are especially unlikely to engage in PA and tend to have poorer health outcomes (16). As a systematic review indicated, ethnic differences should also be considered in the relationship between chronic disease and PA (17). However, such studies are scarce, especially for LTPA.

To help fill these gaps, this study aimed to estimate the association between intersystem and intrasystem chronic disease multimorbidity and LTPA by ethnicities based on baseline data from the China Multi-Ethnic Cohort (CMEC) (18). Our study can help clinicians to better understand the levels of LTPA among different multimorbidity groups within Han and ethnic minorities groups, for better management of chronic disease and multimorbidity.

## Materials and methods

### Study design

This study was a cross-sectional study based on the baseline survey of a community population-based prospective observational study established in five provinces (Sichuan, Guizhou, Yunnan, Chongqing, and Tibet) of southwestern China (the CMEC). Seven ethnic groups were identified based on their census register, namely, the Han, Bouyei, Tibetan, Miao, Bai, Yi, and Dong ethnic groups. In the baseline survey, a total of 99,556 participants aged 30–79 years old were recruited from May 2018 to September 2019 by a multistage, stratified cluster sampling method. The details of the CMEC study design, survey methods, and inclusion criteria for participants have been reported previously (18).

For the current analyzes, we excluded (1) those with severe diseases or conditions that might affect participants’ PA; (2) those with extreme body mass index (BMI), namely, a calculated value > 40 or < 15; (3) those who had both PA and sedentary leisure time equal to zero; and (4) those with missing information on any outcome, exposure, or adjusted covariates. Ultimately, a total of 76,084 participants remained in the analyzes (Supplementary Figure 1).

### Exposures

Studies have shown that the relationship between low PA and multimorbidity is bidirectional (10, 19, 20). Also, a few studies examining multimorbidity have treated chronic diseases as exposure (9, 21). Thus, multimorbidity, including 17 chronic diseases, was treated as exposure in our studies, representing the most common chronic disease among the Chinese population (22). Among these chronic diseases, definitions of hypertension, chronic bronchitis, emphysema, gallstone, cholecystitis, diabetes, and hyperlipidemia were based on both the questionnaires and medical examinations; definitions of pulmonary heart disease, rheumatic heart disease, coronary heart disease, rheumatoid arthritis, asthma, cirrhosis, gastroenteritis, and peptic ulcer were only based on the questionnaire; and definitions of obesity and osteoporosis were only based on medical examinations.

Specific definitions for some chronic diseases were as follows. (1) Hypertension. Participants’ blood pressure was measured three times using the OMROM HEM-8771 monitor (Omron [China] Co., Ltd., Shanghai, China). Hypertension was defined as a mean systolic blood pressure ≥ 140 mm Hg and/or mean diastolic blood pressure ≥ 90 mm Hg and/or a diagnosis of hypertension by doctors (23). (2) Diabetes. Fasting plasma glucose (FPG) and glycosylated hemoglobin (HbA1c) were measured in plasma enzymatically with a validated autoanalyzer (AU5800 Automated Chemistry Analyzer, Beckman Colter Commercial Enterprise, Shanghai, China). Diabetes was defined as FPG ≥ 126 mg/dl and/or HbA1c ≥ 6.5% and/or a diagnosis of diabetes by doctors (24). (3) Hyperlipidemia. Hyperlipidemia was defined based on the presence of one or more of the following components according to the Joint Committee for Developing Chinese Guidelines on Prevention and Treatment of Dyslipidemia in Adults: triglyceride ≥ 2.3 mmol/L, total cholesterol ≥ 6.2 mmol/L, high-density lipoprotein cholesterol < 1.0 mmol/L, low-density lipoprotein cholesterol ≥ 4.1 mmol/L, and/or diagnosis of hyperlipidemia by doctors. (4) Obesity. Height and weight were measured in participants wearing light clothing and barefoot using a weight scale, and BMI was calculated as the body weight (kg) divided by the height squared (m^2^). Obesity was defined as having a BMI ≥ 28.00, according to China’s BMI criterion (25). (5) Osteoporosis. The mineral density of the anklebone was measured using an OSTEOKJ3000 ultrasonic bone densitometer, and a T-score of ≤ −2.5 was defined as osteoporosis (26).

Chronic disease multimorbidity was defined as having at least two of the defined chronic diseases (9). According to the International Classification of Diseases, Tenth Revisions (ICD-10), 17 chronic diseases in this study were organized into four chronic disease subgroups (i.e., diseases of the circulatory system, digestive system, respiratory system, and metabolic system) (Supplementary Table 1). According to the subgroups, multimorbidity was divided into two categories: (1) intrasystem multimorbidity, referred to as multimorbidity within the same system, including circulatory, respiratory, digestive, and metabolic system multimorbidity; and (2) intersystem multimorbidity, referred to as multimorbidity between two systems, including circulatory-respiratory, circulatory-digestive, circulatory-metabolic, respiratory-digestive, respiratory-metabolic, and digestive-metabolic system multimorbidity. This division helps to comprehensively capture the associations of LTPA with either chronic disease multimorbidity or special chronic disease multimorbidity subgroups. In the current study, each type of multimorbidity was treated as a separate exposure.

### Outcomes

The questions on PA and LTPA were adapted from validated questionnaires used in several other studies, namely, the European Prospective Investigation into Cancer and Nutrition (27) and the China Kadoorie Biobank study (28). PA considered participants’ occupational, household, transport, and leisure time (28). All of the participants were asked to report their usual type of LTPA (e.g., walking and jogging) and the average duration spent on LTPA every week over the past year. For a specific LTPA, the second update of metabolic equivalent tasks (METs) from the 2011 Compendium of Physical Activities was used to estimate how many calories are burned (29). The MET values were assigned to each type of LTPA: 3.5 METs for light LTPA (e.g., Taichi, Qigong, and walking), 4.5 METs for moderate LTPA (e.g., jogging and aerobic dancing), 6.0 METs for medium vigorous LTPA (e.g., ballgames and equipment sports), and 7.0 METs for high vigorous LTPA (e.g., swimming). The volume of LTPA (MET-h/week) was calculated by multiplying the intensity (METs) by duration (h/week) (30). According to the minimum level of LTPA recommended by the WHO, those with a volume of LTPA < 7.5 MET-hours/week were defined as low LTPA. In addition, those with a volume of PA less than the median were defined as having low PA (31).

### Statistical analyzes

Descriptive statistics were used for sample characteristics (i.e., demographics and behavior characteristics) under a specific LTPA and ethnicity in Table 1. Continuous and categorical characteristic variables between subjects engaged in low and adequate LTPA were presented as mean ± SD and numbers (percentages) and were compared using Student’s *t*-test and chi-squared test. An intensity matrix was used to represent the linkages between different types of multimorbidity.

**TABLE 1:** Sample characteristics by low or high leisure-time physical activity (LTPA) and ethnicity.

Variables	Han population (*n* = 44,025)			Minority population (*n* = 32,059)		
	High LTPA (*n* = 20,139)	Low LTPA (*n* = 23,886)	*P* -value	High LTPA (*n* = 8,105)	Low LTPA (*n* = 23,954)	*P* -value
Sex (%)			<0.001			0.045
Male	8,346 (41.4)	10,479 (43.9)		2,745 (33.9)	7,822 (32.7)	
Female	11,793 (58.6)	13,407 (56.1)		5,360 (66.1)	16,132 (67.3)	
Age (years) (SD)	52.65 (11.81)	49.46 (11.11)	<0.001	53.58 (11.45)	50.79 (10.92)	<0.001
Marital status (%)			0.001			<0.001
Married/cohabiting	17,951 (89.1)	21,528 (90.1)		7,037 (86.8)	21,396 (89.3)	
Unmarried/divorced/widowed	2,188 (10.9)	2,358 (9.9)		1,068 (13.2)	2,558 (10.7)	
Annual family income, yuan/year (%)			<0.001			<0.001
<20,000	4,315 (21.4)	7,192 (30.1)		3,076 (38.0)	12,435 (51.9)	
20,000–59,999	7,321 (36.4)	9,104 (38.1)		2,937 (36.2)	8,263 (34.5)	
≥ 60,000	8,503 (42.2)	7,590 (31.8)		2,092 (25.8)	3,256 (13.6)	
Education level (%)			<0.001			<0.001
Illiteracy	2,142 (10.6)	3,800 (15.9)		2,946 (36.3)	11,127 (46.5)	
Primary school	3,959 (19.7)	6,666 (27.9)		2,052 (25.3)	6,531 (27.3)	
Junior high school	6,505 (32.3)	7,173 (30.0)		1,595 (19.7)	4,279 (17.9)	
High school or more	7,533 (37.4)	6,247 (26.2)		1,512 (18.7)	2,017 (8.4)	
Smoking status (%)			<0.001			<0.001
Never	14,876 (73.9)	16,876 (70.7)		6,630 (81.8)	19,252 (80.4)	
Former	1,379 (6.8)	1,020 (4.3)		376 (4.6)	715 (3.0)	
Current	3,884 (19.3)	5,990 (25.1)		1,099 (13.6)	3,987 (16.6)	
Alcohol drinking status (%)			<0.001			0.025
Never	10,140 (50.4)	12,620 (52.8)		5,413 (66.8)	15,722 (65.6)	
Occasionally	7,091 (35.2)	7,444 (31.2)		2,021 (24.9)	6,021 (25.1)	
Often	2,908 (14.4)	3,822 (16.0)		671 (8.3)	2,211 (9.2)	
Non- LTPA (MET/day) (SD)	17.01 (14.37)	28.14 (18.81)	<0.001	19.53 (16.78)	29.31 (19.48)	<0.001
Sleep duration (h/day)			<0.001			<0.001
<6	2,190 (10.9)	2,423 (10.1)		976 (12.0)	2,884 (12.0)	
6–8	15,671 (77.8)	18,195 (76.2)		5,427 (67.0)	15,370 (64.2)	
>8	2,278 (11.3)	3,268 (13.7)		1,702 (21.0)	5,700 (23.8)	
Region (%)			<0.001			<0.001
Han ethnicity in Basin	17,697 (87.9)	17,274 (72.3)		–	–	
Han ethnicity in Yunnan	2,442 (12.1)	6,612 (27.7)		–	–	
Yi ethnicity in Yunnan	–	–		877 (10.8)	4,048 (16.9)	
Bai ethnicity in Yunnan	–	–		1,734 (21.4)	3,544 (14.8)	
Tibetans in Aba	–	–		972 (12.0)	2,389 (10.0)	
Tibetans in Lhasa	–	–		1,002 (12.4)	2,502 (10.4)	
Dong ethnicity in Guizhou	–	–		1,301 (16.1)	4,423 (18.5)	
Bouyei ethnicity in Guizhou	–	–		1,269 (15.7)	3,653 (15.3)	
Miao ethnicity in Guizhou	–	–		950 (11.7)	3,395 (14.2)	

SD, standard deviation; LTPA, leisure-time physical activity. The differences in dichotomous (i.e., sex) and polytomous (i.e., region) qualitative variables between subjects engaged in low and high LTPA were tested by the chi-squared test. The differences between quantitative variables were compared by Student’s *t*-test.

Multivariate logistic regression models were used to assess the associations among chronic diseases, multimorbidity, intrasystem multimorbidity, intersystem multimorbidity, and LTPA among the Han and minority populations. The stratified analyzes were used to explore the associations between multimorbidity and LTPA by ethnic group, and the likelihood ratio test was used to examine the significance. The analyzes were adjusted for sex (male or female), age (continuous), marital status (married/cohabiting and unmarried/divorced/widowed), annual family income (3 categories: < 20,000 yuan, 20,000–59,999 yuan, and ≥ 60,000 yuan), educational level (4 categories: illiteracy, primary school, junior high school, and high school or more), smoking (3 categories: never, former, and current), alcohol drinking (3 categories: never, occasionally, and often), sleep duration (3 categories: < 6 hours, 6–8 hours, and > 8 hours), region (9 categories: Han ethnicity in Basin, Han ethnicity in Yunnan, Yi ethnicity in Yunnan, Bai ethnicity in Yunnan, Tibetans in Aba, Tibetans in Lhasa, Dong ethnicity in Guizhou, Bouyei ethnicity in Guizhou, and Miao ethnicity in Guizhou), and non-LTPA (continuous). The number of hours spent per week on each activity was multiplied by the MET score for the activity, and the weekly amount of non-LTPA was obtained by totaling the MET-hours for activities related to occupation, household, and transport except for LTPA. We also detected the overall interaction by including an interaction term reflecting multimorbidity × ethnic group.

In the sensitivity analysis, we adjusted the exposure criteria to the self-reported chronic diseases aforementioned. Although both osteoporosis and obesity were diagnosed by medical examinations, we did not exclude obesity because it was easily perceived by the participants; osteoporosis was excluded since it was diagnosed by medical examination without self-reported data. Finally, we conducted the sensitivity analysis only on multimorbidity for 16 self-reported chronic diseases. The effect estimates were expressed as odds ratios (ORs) and 95% CIs. All of the statistical analyzes were performed using R software, version 4.0.2 (R Foundation for Statistical Computing). A two-tailed *P*-value < 0.05 was declared statistically significant.

## Results

The analytic sample consisted of 76,084 participants aged 30–79 years old. Overall, the mean (SD) age was 51.2 (11.4) years old, 29,392 (38.6%) were men, and 32,059 (42.1%) were minorities. In brief, more than half (62.9%) of the participants engaged in a low level of LTPA, and the minority population (74.7%) was significantly larger than the Han ethnic population (54.3%). For minorities, participants engaged in low LTPA were more likely to be women who were younger, with low annual family income, low educational levels, current smoking, cohabiting, often drinking, high levels of non-LTPA, and long sleep durations. However, for the Han ethnic population, low LTPA tended to appear in men with the aforementioned characteristics (Table 1).

**FIGURE 1:**
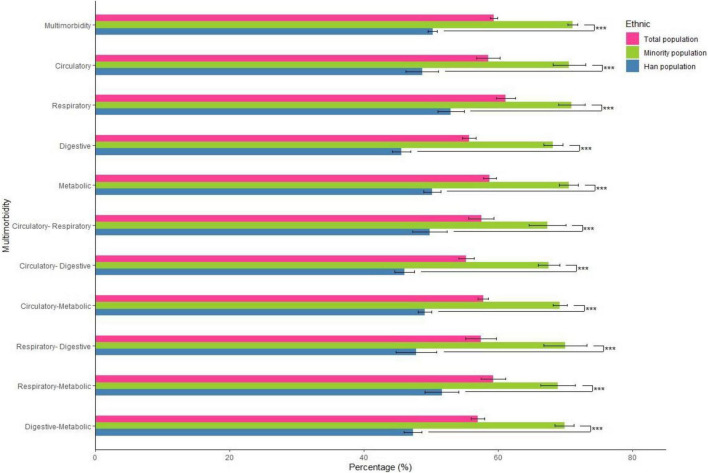
Prevalence of low leisure-time physical activity in participants with each type of multimorbidity by ethnic group. ***p < 0.001.

The prevalence of low LTPA in participants with each type of multimorbidity by ethnic group is shown in Figure 1. In brief, the prevalence of low LTPA among minorities with multimorbidity was significantly higher than that of the Han population. The ORs and 95% CIs for the associations of low LTPA with 17 chronic diseases are shown in Supplementary Table 2.

**FIGURE 2:**
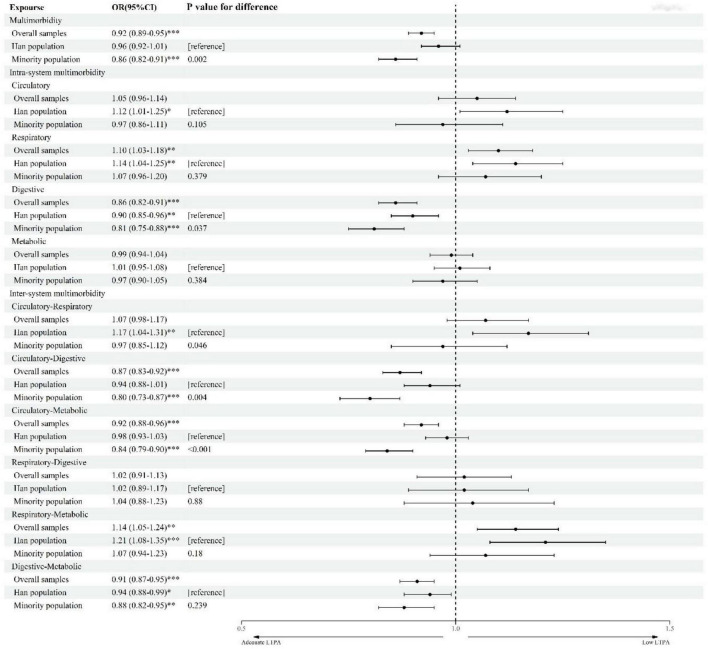
ORs for low leisure-time physical activity associated with chronic disease multimorbidity. Low leisure-time physical activity (LTPA) (<7.5 MET-h/week). Each type of multimorbidity was treated as a separate exposure. All models were adjusted for sex, age, marital status, annual family income, educational level, smoking, alcohol drinking, sleep duration, region, and non-LTPA. Multimorbidity refers to the coexistence of two or more chronic diseases. Intrasystem multimorbidity refers to multimorbidity within the same system. Intersystem multimorbidity refers to multimorbidity between two systems. The likelihood ratio test was used to examine the significance. OR, odds ratio; CI, confidence interval. ^***^p < 0.001; ^**^p < 0.01; *p < 0.05.

The results of the multivariable logistic regression analysis assessing the associations of multimorbidity, intrasystem multimorbidity, and intersystem multimorbidity with low LTPA are presented in Figure 2. In the overall sample, we observed that respiratory system multimorbidity (OR = 1.10, 95% CI = 1.03–1.18) and respiratory-metabolic system multimorbidity (OR = 1.14, 95% CI = 1.05–1.24) were associated with an increased risk of low LTPA in the adjusted models, while significantly negative associations were observed for multimorbidity (OR = 0.92, 95% CI = 0.89–0.95), digestive system multimorbidity (OR = 0.86, 95% CI = 0.82–0.91), circulatory-digestive system multimorbidity (OR = 0.87, 95% CI = 0.83–0.92), circulatory-metabolic system multimorbidity (OR = 0.92, 95% CI = 0.88–0.96), and digestive-metabolic system multimorbidity (OR = 0.91, 95% CI = 0.87–0.95). Notably, when we considered total PA (Supplementary Table 3), we found that all types of multimorbidity were positively associated with low PA. The intensity matrix suggested some types of multimorbidity (i.e., respiratory and respiratory-digestive) were closely associated (Supplementary Figure 2). The overall interaction between multimorbidity and ethnic group was not significant (OR = 0.95, 95%CI = 0.89–1.02).

When the analysis was stratified by the ethnic group, we found some interesting results (Figure 2). Minority population suffered from multimorbidity tended to have adequate LTPA (OR = 0.86, 95% CI = 0.82–0.91), while a significant association was not found among the Han population (OR = 0.96, 95% CI = 0.92–1.01). In addition, for intrasystem multimorbidity and intersystem multimorbidity, all significant associations with low LTPA were negative in the minority population but were mainly positive in the Han population. For both the minority and Han populations, digestive system multimorbidity and digestive-metabolic system multimorbidity had negative, significant associations with low LTPA. For the Han population, the association of circulatory-respiratory system multimorbidity (OR = 1.17, 95% CI = 1.04–1.31) with low LTPA was stronger than those of circulatory system multimorbidity (OR = 1.12, 95% CI = 1.01–1.25) and respiratory system multimorbidity (OR = 1.14, 95% CI = 1.04–1.25).

The sensitivity analyzes suggested similar effect estimates for low LTPA (Supplementary Table 4). Compared to the corresponding OR in Figure 2, for the Han population, the OR in the association between low LTPA and intrasystem multimorbidity of circulatory, respiratory, and circulatory-respiratory system multimorbidity increased; for minorities, the OR in the associations of low LTPA with circulatory system multimorbidity, circulatory-metabolic system multimorbidity, and digestive-metabolic system multimorbidity decreased.

## Discussion

This study examined the associations of multimorbidity, intrasystem multimorbidity, and intersystem multimorbidity with low LTPA among Han and minority populations based on a large-scale, multiethnic cohort in China. Our findings revealed people with chronic disease multimorbidity were inclined to engage in more LTPA in the overall sample, and the association in minority populations was stronger than that in the Han population. In addition, the ethnic differences in the associations between multimorbidity and low LTPA were not found as the interaction between multimorbidity and ethnic group was not significant. These findings could enable clinicians and policymakers to carry out precise PA interventions and treatments for those with particular chronic disease multimorbidity.

To date, many studies have demonstrated that people with chronic diseases or multimorbidity tend to engage in less PA (21, 32–34). However, evidence about the association of chronic disease multimorbidity with LTPA has been relatively scarce. Considering that LTPA is largely modifiable, our study focused on LTPA and observed people with multimorbidity tend to engage in more LTPA, which could provide important evidence for chronic disease multimorbidity control. Notably, our finding is inconsistent with those of the studies aforementioned (21, 32–34). The most likely reason could be that our outcome variable is not the same as theirs, and we only focused on one domain of PA. As shown in Supplementary Table 3, we found that every multimorbidity was positively associated with low PA when considering PA related to occupation, household, transport, and leisure time as the outcome variable. In addition, a person living with multimorbidity may be recommended by clinicians to intentionally engage in more LTPA. Similar to our results, a negative association between chronic diseases and the different domains of PA was found in a study in Austria involving 8,251 participants (35). Our findings proved again that it is necessary to explore the relationships between different domains of PA and multimorbidity.

There are some interesting findings in the association between multimorbidity and low LTPA by the ethnic group. Among minority populations with multimorbidity, digestive system multimorbidity, digestive-metabolic, circulatory-metabolic, or circulatory-digestive system multimorbidity took part in more LTPA. As shown in Supplementary Table 4, the ORs for most multimorbidity decreased compared with Figure 2 among minorities, suggesting that minorities with multimorbidity were likely to engage in adequate LTPA when they have learned about their body status. In addition, people might not expect digestive-metabolic system disorders to limit activity (10). Conversely, LTPA can contribute to the therapy of chronic diseases, leading to a higher level of LTPA in the population with chronic diseases (36). For the Han population with respiratory system multimorbidity or circulatory system multimorbidity, their LTPA levels were lower. As shown in Supplementary Table 4, the ORs for all types of multimorbidity increased compared with Figure 2 except for respiratory-metabolic system multimorbidity among the Han population. One reason could be that the disease might be directly responsible for lower activity levels due to a reduced exercise capacity by influencing cardiopulmonary function (37). A UK Biobank study of 52,556 participants reported that people with the worst cardiometabolic diseases performed approximately half of moderate to vigorous PA on a daily basis compared to healthy individuals (38). Alternatively, physical inactivity leads to an increased risk of these diseases, and individuals might have been habitually less active than their healthy counterparts for some time to develop the condition (10). Participants with lower LTPA had a greater likelihood of developing chronic diseases, such as metabolic syndrome (39). Moreover, a cohort study conducted for 3 years found that the number of patients with metabolic syndrome having sufficient PA did not significantly increase despite advice to increase PA (40). Multimorbidity can contribute to dementia and cognitive decline (41), which could influence LTPA. For digestive system multimorbidity and digestive-metabolic system multimorbidity among the Han population, their associations with LTPA could be attributed to the same reasons aforementioned in the minority population. The association between chronic conditions or multimorbidity and LTPA might differ in ethnicities due to different disease profiles (42), suboptimal treatment of chronic conditions (43, 44), differences in knowledge regarding the benefits of PA (45), or other environmental factors, such as work conditions (46). Han populations with multimorbidity tended to report low LTPA in this study, contradicting our current knowledge that ethnic minorities engage in less healthy behaviors than the Han people. More future studies are warranted to verify such findings.

We observed that intersystem multimorbidity has a possible synergistic effect on LTPA. For example, among the overall sample, the ORs for low LTPA with respiratory system multimorbidity and metabolic system multimorbidity were 1.10 and 0.99, respectively. However, the effect values increased to 1.14 in the association between low LTPA and respiratory-metabolic system multimorbidity. Consistent synergistic effects among circulatory-respiratory system multimorbidity, respiratory-metabolic system, and low LTPA were also observed among the Han population. The effect has some biological plausibility, with the consideration of several pathways. First, shortness of breath caused by chronic respiratory disease might be worse due to the additional influence of chronic circulatory disease. It seems to be difficult for patients to engage in sufficient LTPA owing to breathing problems (47). Second, the connectedness of several chronic diseases might increase symptom severity, especially pain perception, which could in turn explain why patients with chronic respiratory disease and other chronic diseases avoid LTPA (48). Finally, there is evidence that diseases belonging to common patterns of multimorbidity can interact, curtailing compensatory mechanisms and resulting in more severe physical inactivity (37).

As is known to all, limitations in physical and cognitive function due to multimorbidity decisively reduce people’s levels of PA, which can in turn increase multimorbidity (19, 20). However, our study found people suffering from multimorbidity may engage in adequate LTPA, which suggested that clinicians should not blindly advise patients with multimorbidity to do physical exercise in their spare time.

## Limitations and strengths

Several limitations to this study should be considered. First, eight chronic diseases were self-reported based on the questionnaire, and the results could have been affected by information bias. Second, PA could have been influenced by many factors. Although it was impossible to adjust for all of the potential confounding factors, variables such as green space, the built environment, and cognitive function (41, 49, 50) should have been considered. Third, our research only focused on seventeen chronic diseases with a high prevalence in southwest China and excluded people with special health statuses (e.g., infectious diseases, pregnancy, and injury) that might severely affect PA; other diseases affecting PA were not considered either. Fourth, since this study was a cross-sectional study, caution should be taken in making causal interpretations between LTPA and multimorbidity. Nonetheless, our study has notable strengths. Our findings clarified multimorbidity into intrasystem multimorbidity and intersystem multimorbidity, which helps us comprehensively understand the associations of LTPA with specific multimorbidity subgroups.

## Conclusion

This large-scale epidemiologic study provides an improved understanding of the impact of multimorbidity on LTPA in Han and minority populations in southwest China. Our findings suggest that LTPA-tailored interventions should be designed for specific ethnic groups according to a different type of multimorbidity. Future large, prospective studies are required to further determine the temporality of the associations observed in this investigation and better explain whether changes in the nature of the dependent variable produce different results.

## Data availability statement

The original contributions presented in this study are included in the article/Supplementary material, further inquiries can be directed to the corresponding authors.

## Ethics statement

The studies involving human participants were reviewed and approved by the Ethics Committee of Sichuan University (K2016038). The patients/participants provided their written informed consent to participate in this study.

## Author contributions

YL, QN, and JZ: conceptualization. JL, RH, and QN: data curation. YL, XL, and BY: formal analysis and writing—original draft. YL, QN, JL, and RH: investigation. YL, BY, XL, and JZ: methodology. QN: project administration. JZ, YL, XL, and BY: writing—review & editing. All authors have read and agreed to the published version of the manuscript.

## References

[1] van den Akker M Buntinx F Roos S Knottnerus JA. Problems in determining occurrence rates of multimorbidity. *J Clin Epidemiol.* (2001) 54:675–9. 11438407 10.1016/s0895-4356(00)00358-9

[2] Garin N Koyanagi A Chatterji S Tyrovolas S Olaya B Leonardi M Global multimorbidity patterns: a cross-sectional, population-based, multi-country study. *J Gerontol A Biol Sci Med Sci.* (2016) 71:205–14. 10.1093/gerona/glv128 26419978 PMC5864156

[3] He L Biddle SJH Lee JT Duolikun N Zhang L Wang Z The prevalence of multimorbidity and its association with physical activity and sleep duration in middle aged and elderly adults: a longitudinal analysis from China. *Int J Behav Nutr Phys Act.* (2021) 18:77. 10.1186/s12966-021-01150-7 34112206 PMC8194125

[4] Cassell A Edwards D Harshfield A Rhodes K Brimicombe J Payne R The epidemiology of multimorbidity in primary care: a retrospective cohort study. *Br J Gen Pract.* (2018) 68:e245–51. 29530918 10.3399/bjgp18X695465 PMC5863678

[5] Nicholson K Almirall J Fortin M. The measurement of multimorbidity. *Health Psychol.* (2019) 38:783–90. 31021126 10.1037/hea0000739

[6] Kushner RF Sorensen KW. Lifestyle medicine: the future of chronic disease management. *Curr Opin Endocrinol Diabetes Obes.* (2013) 20:389–95. 23974765 10.1097/01.med.0000433056.76699.5d

[7] Anderson E Durstine JL. Physical activity, exercise, and chronic diseases: a brief review. *Sports Med Health Sci.* (2019) 1:3–10. 35782456 10.1016/j.smhs.2019.08.006 PMC9219321

[8] Abegunde DO Mathers CD Adam T Ortegon M Strong K. The burden and costs of chronic diseases in low-income and middle-income countries. *Lancet.* (2007) 370:1929–38. 18063029 10.1016/S0140-6736(07)61696-1

[9] Vancampfort D Koyanagi A Ward PB Rosenbaum S Schuch FB Mugisha J Chronic physical conditions, multimorbidity and physical activity across 46 low- and middle-income countries. *Int J Behav Nutr Phys Act.* (2017) 14:6. 10.1186/s12966-017-0463-5 28100238 PMC5241915

[10] Barker J Smith Byrne K Doherty A Foster C Rahimi K Ramakrishnan R Physical activity of UK adults with chronic disease: cross-sectional analysis of accelerometer-measured physical activity in 96 706 UK Biobank participants. *Int J Epidemiol.* (2019) 48:1167–74. 30721947 10.1093/ije/dyy294 PMC6693885

[11] Holtermann A Krause N van der Beek A Straker L. The physical activity paradox: six reasons why occupational physical activity (OPA) does not confer the cardiovascular health benefits that leisure time physical activity does. *Br J Sports Med.* (2018) 52:149. 10.1136/bjsports-2017-097965 28798040

[12] Massucci M Perrero L Mantellini E Petrozzino S Gamna F Nocella A Cardiorespiratory comorbidity: a new challenge for physical and rehabilitation medicine specialist. *Eur J Phys Rehabil Med.* (2012) 48:1–8. 21750484

[13] Dagar A Falcone T. Psychiatric comorbidities in pediatric epilepsy. *Curr Psychiatry Rep.* (2020) 22:77. 10.1007/s11920-020-01195-8 33128638

[14] Gay JL Buchner DM. Ethnic disparities in objectively measured physical activity may be due to occupational activity. *Prev Med.* (2014) 63:58–62. 24589439 10.1016/j.ypmed.2014.02.015

[15] Sternfeld B Colvin A Stewart A Appelhans BM Cauley JA Dugan SA Understanding racial/ethnic disparities in physical performance in midlife women: findings from SWAN (Study of women’s health across the nation). *J Gerontol B Psychol Sci Soc Sci.* (2020) 75:1961–71. 10.1093/geronb/gbz103 31412129 PMC7566973

[16] Miller LS Gramzow RH. A self-determination theory and motivational interviewing intervention to decrease racial/ethnic disparities in physical activity: rationale and design. *BMC Public Health.* (2016) 16:768. 10.1186/s12889-016-3413-2 27515173 PMC4982425

[17] Gill JM Celis-Morales CA Ghouri N. Physical activity, ethnicity and cardio-metabolic health: does one size fit all?. *Atherosclerosis.* (2014) 232:319–33. 10.1016/j.atherosclerosis.2013.11.039 24468145

[18] Zhao X Hong F Yin J Tang W Zhang G Liang X Cohort Profile: the China Multi-Ethnic Cohort (CMEC) study. *Int J Epidemiol.* (2021) 50:721–721l. 10.1093/ije/dyaa185 33232485 PMC8271196

[19] Parsons S Gale CR Kuh D Elliott J The Halcyon Study Team. Physical capability and the advantages and disadvantages of ageing: perceptions of older age by men and women in two British cohorts. *Ageing Soc.* (2014) 34:452–71.

[20] Ryan A Wallace E O’Hara P Smith SM. Multimorbidity and functional decline in community-dwelling adults: a systematic review. *Health Qual Life Outcomes.* (2015) 13:13. 10.1186/s12955-015-0355-9 PMC4606907 26467295

[21] Keats MR Cui Y DeClercq V Dummer TJB Forbes C Grandy SA Multimorbidity in Atlantic Canada and association with low levels of physical activity. *J Prev Med.* (2017) 105:326–31. 10.1016/j.ypmed.2017.10.013 28987335

[22] Wang HH Wang JJ Wong SY Wong MC Li FJ Wang PX Epidemiology of multimorbidity in China and implications for the healthcare system: cross-sectional survey among 162,464 community household residents in southern China. *BMC Med.* (2014) 12:188. 10.1186/s12916-014-0188-0 25338506 PMC4212117

[23] Lewington S Lacey B Clarke R Guo Y Kong XL Yang L The burden of hypertension and associated risk for cardiovascular mortality in China. *JAMA Intern Med.* (2016) 176:524–32. 26975032 10.1001/jamainternmed.2016.0190

[24] American Diabetes Association. Diagnosis and classification of diabetes mellitus. *Diabetes Care.* (2013) 36:S67–74. 23264425 10.2337/dc13-S067 PMC3537273

[25] Limin W Bin Z Zhenping Z Yang L Zhang M Jiang Y Body-mass index and obesity in urban and rural China: findings from consecutive nationally representative surveys during 2004-18. *J Lancet.* (2021) 398:53–63. 10.1016/S0140-6736(21)00798-4 34217401 PMC7617101

[26] Hammond I Lyons DJ. Bone mineral densitometry reporting and the CAR technical standards: tips for the radiologist. *Can Assoc Radiol J.* (2020) 71:134–5. 10.1177/0846537119899270 32063013

[27] Wareham NJ Jakes RW Rennie KL Mitchell J Hennings S Day NE. Validity and repeatability of the EPIC-Norfolk physical activity questionnaire. *Int J Epidemiol.* (2002) 31:168–74. 10.1093/ije/31.1.168 11914316

[28] Du H Bennett D Li L Whitlock G Guo Y Collins R Physical activity and sedentary leisure time and their associations with BMI, waist circumference, and percentage body fat in 0.5 million adults: the China Kadoorie Biobank study. *Am J Clin Nutr.* (2013) 97:487–96. 10.3945/ajcn.112.046854 23364014 PMC4345799

[29] Ainsworth B Haskell W Herrmann S Meckes N Bassett DR Jr. Tudor-Locke C 2011 Compendium of Physical Activities: a second update of codes and MET values. *Med Sci Sports Exerc.* (2011) 43:1575–81. 10.1249/MSS.0b013e31821ece12 21681120

[30] Lao XQ Deng HB Liu X Chan TC Zhang Z Chang LY Increased leisure-time physical activity associated with lower onset of diabetes in 44 828 adults with impaired fasting glucose: a population-based prospective cohort study. *Br J Sports Med.* (2019) 53:895–900. 10.1136/bjsports-2017-098199 29331993

[31] Yajie L Qucuo N Bin Y Xiao X Zeng P Suolang D Determinants of self-rated health among an older Tibetan population in a Chinese plateau area: analysis based on the conceptual framework for determinants of health. *J BMC Public Health.* (2021) 21:489. 10.1186/s12889-021-10359-x 33706725 PMC7953750

[32] Shen G Zhang Y Bo X Wu W Li X Yu X The status and influencing factors of physical activity among patients with chronic kidney disease. *J Nurs Sci*. (2019) 34:25–29.

[33] Castillo SS Smith L Suárez A Sánchez G. Physical activity behaviour in people with COPD residing in Spain: a cross-sectional analysis. *Lung.* (2019) 197:3. 10.1007/s00408-019-00287-4 31686208

[34] Loprinzi P. Accelerometer-determined sedentary and physical activity estimates among older adults with diabetes: considerations by demographic and comorbidity characteristics. *J Aging Phys Act.* (2014) 22:432–40. 10.1123/japa.2013-0019 24084172

[35] Ernst DT Christian L Sandra H Igor G Viktoria SK. The influence of occupational categories on overall and domain-specific physical activity and the association with chronic diseases. An analysis using the Austrian health interview survey. *Int J Environ Res Public Health.* (2021) 18:2148. 10.3390/ijerph18042148 33671784 PMC7926308

[36] Haskell WL Lee IM Pate RR Powell KE Franklin BA. Physical activity and public health: updated recommendation for adults from the American college of sports medicine and the American heart association. *Med Sci Sports Exerc.* (2007) 1116:1081–93. 10.1161/CIRCULATIONAHA.107.185649 17671237

[37] Calderón-Larrañaga A Vetrano DL Ferrucci L Mercer SW Marengoni A Onder G Multimorbidity and functional impairment: bidirectional interplay, synergistic effects and common pathways. *J Intern Med.* (2018) 285:255–71. 10.1111/joim.12843 30357990 PMC6446236

[38] Cassidy S Fuller H Chau J Catt M Bauman A Trenell MI. Accelerometer-derived physical activity in those with cardio-metabolic disease compared to healthy adults: a UK Biobank study of 52,556 participants. *Acta Diabetol.* (2018) 55:975–9. 10.1007/s00592-018-1161-8 29808390 PMC6096713

[39] Turi BC Codogno JS Fernandes RA Monteiro HL. Low levels of physical activity and metabolic syndrome: cross-sectional study in the Brazilian public health system. *Cien Saude Colet.* (2016) 21:1043–50. 10.1590/1413-81232015214.23042015 27076003

[40] Hanneke J den Engelsen C Rutten GE. Physical activity in patients with metabolic syndrome: at screening and three years thereafter. *Metab Syndr Relat Disord.* (2013) 11:163–8. 10.1089/met.2012.0110 23438154

[41] Koroukian S Schiltz N Warner D Sun J Bakaki PM Smyth KA Combinations of Chronic Conditions, Functional Limitations, and Geriatric Syndromes that Predict Health Outcomes. *J Gen Intern Med.* (2016) 31:630–7. 26902246 10.1007/s11606-016-3590-9 PMC4870414

[42] Forouzanfar MH Alexander L Anderson HR Bachman VF Biryukov S Brauer M Global, regional, and national comparative risk assessment of 79 behavioural, environmental and occupational, and metabolic risks or clusters of risks in 188 countries, 1990-2013: a systematic analysis for the Global Burden of Disease Study 2013. *Lancet.* (2015) 386:2287–323. 26364544 10.1016/S0140-6736(15)00128-2 PMC4685753

[43] Patel V Araya R Chatterjee S Chisholm D Cohen A De Silva M Treatment and prevention of mental disorders in low-income and middle-income countries. *Lancet.* (2007) 370:991–1005. 17804058 10.1016/S0140-6736(07)61240-9

[44] Chow CK Teo KK Rangarajan S Islam S Gupta R Avezum A Prevalence, awareness, treatment, and control of hypertension in rural and urban communities in high-, middle-, and low-income countries. *JAMA.* (2013) 310:959–68. 24002282 10.1001/jama.2013.184182

[45] Pengpid S Peltzer K Kassean HK Tsala Tsala JP Sychareun V Müller-Riemenschneider F. Physical inactivity and associated factors among university students in 23 low-, middle- and high-income countries. *Int J Public Health.* (2015) 60:539–49. 10.1007/s00038-015-0680-0 25926342

[46] Atkinson K Lowe S Moore S. Human development, occupational structure and physical inactivity among 47 low and middle income countries. *Prev Med Rep.* (2016) 3:40–5. 10.1016/j.pmedr.2015.11.009 26844185 PMC4733059

[47] Dörenkamp S Mesters I Vos R Schepers J van den Akker M Teijink J Synergistic effects of six chronic disease pairs on decreased physical activity: the SMILE cohort study. *BioMed Res Int.* (2016) 2016:9427231. 10.1155/2016/9427231 27274994 PMC4871954

[48] Sarah D Ilse M Rein V Schepers J van den Akker M Teijink J Synergistic effects of six chronic disease pairs on decreased physical activity: the SMILE cohort study. *J BioMed Res Int.* (2016) 2016:9427231. 10.1155/2016/9427231 27274994 PMC4871954

[49] Klompmaker J Janssen N Bloemsma L Gehring U Wijga AH van den Brink C Associations of combined exposures to surrounding green, air pollution, and road traffic noise with cardiometabolic diseases. *Environ Health Perspect.* (2019) 127:87003. 10.1289/EHP3857 PMC6792364 31393793

[50] Huang W Yang B Yu H Bloom MS Markevych I Heinrich J Association between community greenness and obesity in urban-dwelling Chinese adults. *Science Total Environ.* (2020) 702:135040. 10.1016/j.scitotenv.2019.135040 31726339

