# Supplementary material for: Association between chronic disease multimorbidity and leisure-time physical activity: Evidence from the China Multiethnic Cohort study
Source: Front Med (Lausanne). 2022 Jul 27;9:874456. doi: 10.3389/fmed.2022.874456 (PMC9363590; doi:10.3389/fmed.2022.874456)
Supplement: Supplementary file 1 [file Data_Sheet_1.docx]

**Figure S1. Flow charts for participants enrolment**

**
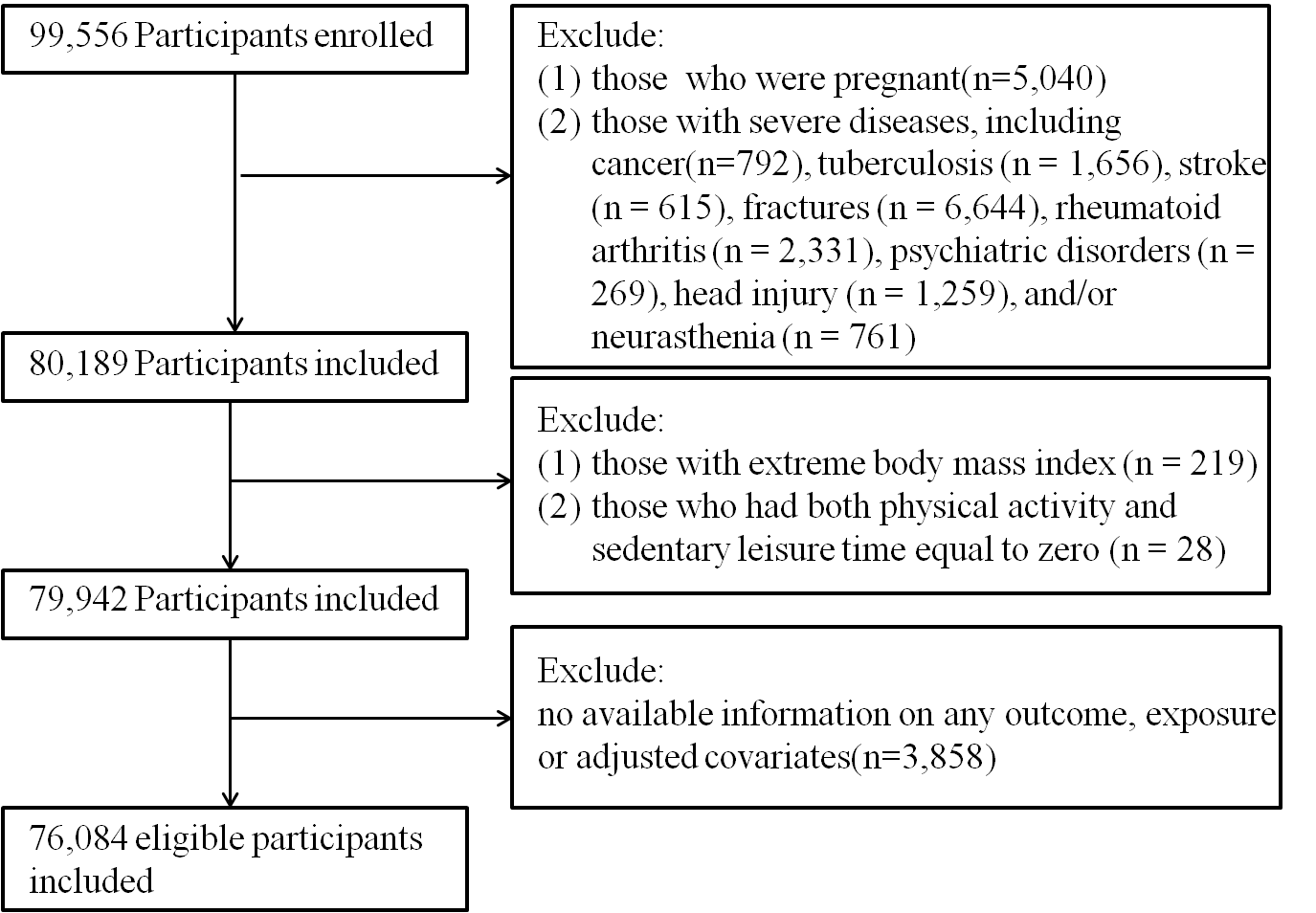
**

**
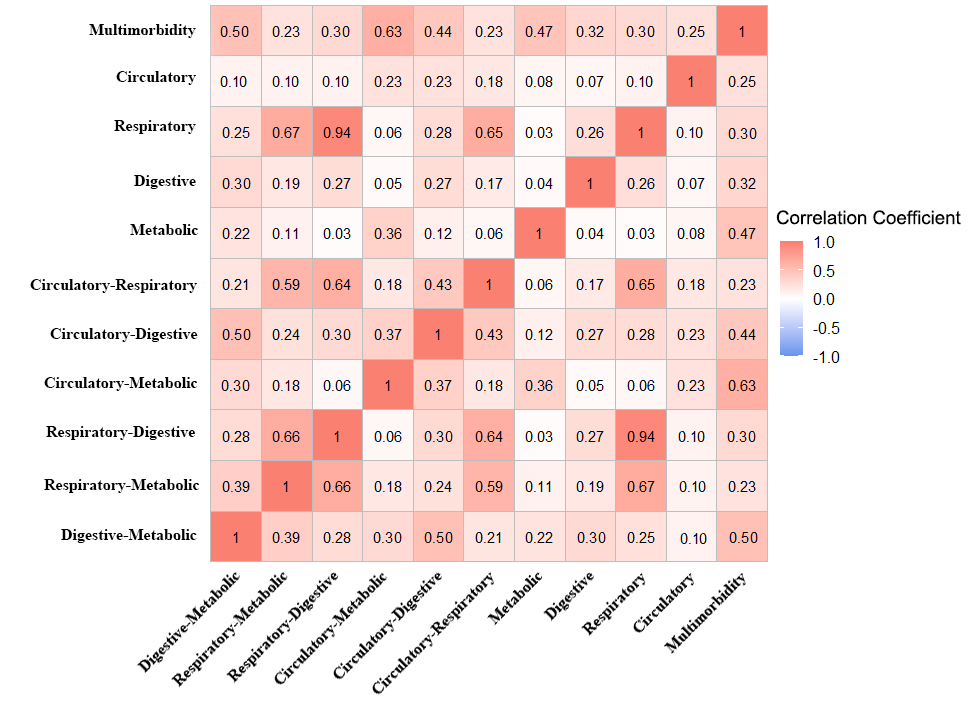
**

**Figure S2. Correlation matrix for different types of multimorbidity**

Notes: all correlation coefficients were statistically significant (p<0.05)

**Table S1. Systematic classification of 17 studied chronic diseases**

| Disease system | Chronic diseases |
| --- | --- |
| Circulatory system | Hypertension, Pulmonary heart disease, Rheumatic heart disease,  Rheumatoid arthritis, Coronary heart disease |
| Digestive system | Gallstones, Peptic ulcer, Cirrhosis, Cholecystitis, Gastroenteritis |
| Respiratory system | Chronic bronchitis, Asthma, Emphysema, |
| Metabolic system | Diabetes, Hyperlipidaemia, Obesity, Osteoporosis |

**Table S2. ORs for low leisure-time physical activity associated with chronic diseases**

|  | Han population  OR (95% CI) | Minority population  OR (95% CI) | P-value |
| --- | --- | --- | --- |
| Hypertension | 0.98 (0.93-1.02) | 0.87 (0.82-0.92) ^***^ | 0.003 |
| Pulmonary heart disease | 1.16 (0.71-1.90) | 1.18 (0.81-1.72) | 0.945 |
| Rheumatic heart disease | 1.19 (0.69-2.07) | 0.53 (029-0.94) ^*^ | 0.046 |
| Coronary heart disease | 1.15 (1.01-1.32) ^*^ | 0.85 (0.70-1.04) | 0.013 |
| Rheumatoid arthritis | 1.07 (0.97-1.18) | 1.00 (0.90-1.12) | 0.391 |
| Chronic bronchitis | 1.13 (1.03-1.23) **^**^** | 1.07 (0.96-1.19) | 0.470 |
| Asthma | 1.24 (1.04-1.49) **^*^** | 0.94 (0.70-1.26) | 0.117 |
| Emphysema | 1.14 (1.05-1.25) **^**^** | 1.08 (0.97-1.20) | 0.403 |
| Gallstones | 0.92 (0.86-0.98) **^**^** | 0.81 (0.76-0.87) ^***^ | 0.014 |
| Peptic ulcer | 0.98 (0.87-1.12) | 1.00 (0.83-1.19) | 0.921 |
| Cirrhosis | 1.06 (0.94-1.19) | 1.05 (0.86-1.27) | 0.929 |
| Cholecystitis | 0.91 (0.85-0.97) **^**^** | 0.82 (0.75-0.88) ^***^ | 0.053 |
| Gastroenteritis | 0.97 (0.91-1.03) | 0.98 (0.90-1.07) | 0.723 |
| Diabetes | 0.92 (0.86-0.98) **^*^** | 0.78 (0.72-0.86) ^***^ | 0.004 |
| Hyperlipidaemia | 0.94 (0.90-0.98) **^**^** | 0.97 (0.92-1.03) | 0.302 |
| Obesity | 1.04 (0.98-1.11) | 0.97 (0.90-1.05) | 0.173 |
| Osteoporosis | 1.28 (1.17-1.40) **^***^** | 1.06 (0.94-1.20) | 0.014 |

Low leisure-time physical activity (LTPA) (< 7.5 MET-hours/week).

Each type of chronic disease was treated as a separate exposure. All models were adjusted for sex, age, marital status, annual family income, educational level, smoking, alcohol drinking, sleep duration, region, and non-LTPA.

The likelihood ratio test was used to examine the statistical significance.

Abbreviations: OR: odds ratio; CI: confidence interval

*p<0.05; **p<0.01; *** p<0.001.

**Table S3. ORs for low physical activity associated with chronic disease multimorbidity**

|  | Total  OR (95% CI) | Han population  OR (95% CI) | Minority population  OR (95% CI) | P-value |
| --- | --- | --- | --- | --- |
| Multimorbidity | 1.22 (1.18-1.26) ^***^ | 1.20 (1.15-1.26) ^***^ | 1.25 (1.19-1.31)^***^ | 0.277 |
| ***Intrasystem multimorbidity*** | | | | |
| Circulatory | 1.16 (1.07-1.26) ^***^ | 1.15 (1.03-1.29) ^*^ | 1.16 (1.03-1.31) ^*^ | 0.932 |
| Respiratory | 1.15 (1.07-1.23) ^***^ | 1.12 (1.02-1.23) ^*^ | 1.18 (1.07-1.31) ^***^ | 0.423 |
| Digestive | 1.19 (1.13-1.25) ^***^ | 1.25 (1.17-1.33) ^***^ | 1.13 (1.05-1.21) ^***^ | 0.047 |
| Metabolic | 1.33 (1.27-1.39) ^***^ | 1.26 (1.19-1.34) ^***^ | 1.41 (1.31-1.52) ^***^ | 0.021 |
| ***Intersystem multimorbidity*** | | | | |
| Circulatory-Respiratory | 1.18 (1.08-1.29) ^***^ | 1.10 (0.97-1.23) | 1.29 (1.13-1.47) ^***^ | 0.076 |
| Circulatory-Digestive | 1.13 (1.07-1.19) ^***^ | 1.12 (1.05-1.21) ^**^ | 1.14 (1.05-1.23) ^**^ | 0.767 |
| Circulatory-Metabolic | 1.24 (1.19-1.28) ^***^ | 1.21 (1.15-1.28) ^***^ | 1.26 (1.19-1.34) ^***^ | 0.318 |
| Respiratory- Digestive | 1.20 (1.08-1.33) ^***^ | 1.28 (1.11-1.47) ^***^ | 1.12 (0.96-1.31) | 0.229 |
| Respiratory-Metabolic | 1.30 (1.19-1.41) ^***^ | 1.26 (1.13-1.42) ^***^ | 1.33 (1.17-1.51) ^***^ | 0.574 |
| Digestive-Metabolic | 1.24 (1.19-1.30) ^***^ | 1.29 (1.21-1.37) ^***^ | 1.19 (1.11-1.28) ^***^ | 0.107 |

Low physical activity (less than the median metabolic equivalent for task (MET) of physical activity).

Each type of multimorbidity was treated as a separate exposure. All models were adjusted for sex, age, marital status, annual family income, educational level, smoking, alcohol drinking, sleep duration, and region.

Multimorbidity refers to the coexistence of two or more chronic diseases. Intrasystem multimorbidity refers to multimorbidity within the same system. Intersystem multimorbidity refers to multimorbidity between two systems.

The likelihood ratio test was used to examine the significance.

Abbreviations: OR: odds ratio; CI: confidence interval.

*p<0.05; **p<0.01; *** p<0.001.

**Table S4. ORs for low leisure-time physical activity associated with chronic diseases multimorbidity in sensitive analysis**

|  | Total  OR (95% CI) | Han population  OR (95% CI) | Minority population  OR (95% CI) | P-value |
| --- | --- | --- | --- | --- |
| Multimorbidity | 0.91 (0.88-0.95) ^***^ | 0.96 (0.91-1.01) | 0.86 (0.81-0.91) ^***^ | 0.007 |
| ***Intrasystem multimorbidity*** | | | | |
| Circulatory | 1.08 (0.98-1.19) | 1.20 (1.05-1.36) ^**^ | 0.95 (0.81-1.10) | 0.021 |
| Respiratory | 1.10 (1.02-1.18) ^*^ | 1.16 (1.06-1.28) ^**^ | 1.03 (0.92-1.15) | 0.100 |
| Digestive | 0.86 (0.82-0.91) ^***^ | 0.90 (0.85-0.96) ^**^ | 0.82 (0.75-0.88) ^***^ | 0.055 |
| Metabolic | 0.89 (0.81-0.97) ^*^ | 0.97 (0.87-1.09) | 0.77 (0.66-0.91) ^**^ | 0.026 |
| ***Intersystem multimorbidity*** | | | | |
| Circulatory-Respiratory | 1.04 (0.93-1.17) | 1.25 (1.08-1.45) ^**^ | 0.84 (0.71-0.99) ^*^ | < 0.001 |
| Circulatory-Digestive | 0.88 (0.82-0.94) ^***^ | 0.94 (0.86-1.02) | 0.82 (0.74-0.92) ^***^ | 0.070 |
| Circulatory-Metabolic | 0.87 (0.82-0.92) ^***^ | 0.92 (0.85-0.99) ^*^ | 0.82 (0.74-0.90) ^***^ | 0.063 |
| Respiratory-Digestive | 1.00 (0.90-1.12) | 1.03 (0.89-1.19) | 1.00 (0.83-1.20) | 0.824 |
| Respiratory-Metabolic | 0.99 (0.88-1.13) | 1.09 (0.93-1.28) | 0.91 (0.74-1.11) | 0.173 |
| Digestive-Metabolic | 0.87 (0.81-0.93) ^***^ | 0.90 (0.83-0.98) ^*^ | 0.84 (0.75-0.94) ^**^ | 0.358 |

Low leisure-time physical activity (LTPA) (< 7.5 MET-hours/week).

Each type of multimorbidity was treated as a separate exposure. All models were adjusted for sex, age, marital status, annual family income, educational level, smoking, alcohol drinking, sleep duration, region, and non-LTPA.

Multimorbidity refers to the coexistence of two or more chronic diseases. Intrasystem multimorbidity refers to multimorbidity within the same system. Intersystem multimorbidity refers to multimorbidity between two systems. The likelihood ratio test was used to examine the significance.

Abbreviations: OR: odds ratio; CI: confidence interval.

*p<0.05; **p<0.01; *** p<0.001.
